# Crimean-Congo hemorrhagic fever virus nucleocapsid protein has dual RNA binding modes

**DOI:** 10.1371/journal.pone.0184935

**Published:** 2017-09-18

**Authors:** Subbiah Jeeva, Sean Pador, Brittany Voss, Safder Saieed Ganaie, Mohammad Ayoub Mir

**Affiliations:** Western University of Health Sciences, Pomona, California, United States of America; Colorado State University, UNITED STATES

## Abstract

Crimean Congo hemorrhagic fever, a zoonotic viral disease, has high mortality rate in humans. There is currently no vaccine for Crimean Congo hemorrhagic fever virus (CCHFV) and chemical interventions are limited. The three negative sense genomic RNA segments of CCHFV are specifically encapsidated by the nucleocapsid protein into three ribonucleocapsids, which serve as templates for the viral RNA dependent RNA polymerase. Here we demonstrate that CCHFV nucleocapsid protein has two distinct binding modes for double and single strand RNA. In the double strand RNA binding mode, the nucleocapsid protein preferentially binds to the vRNA panhandle formed by the base pairing of complementary nucleotides at the 5’ and 3’ termini of viral genome. The CCHFV nucleocapsid protein does not have RNA helix unwinding activity and hence does not melt the duplex vRNA panhandle after binding. In the single strand RNA binding mode, the nucleocapsid protein does not discriminate between viral and non-viral RNA molecules. Binding of both vRNA panhandle and single strand RNA induce a conformational change in the nucleocapsid protein. Nucleocapsid protein remains in a unique conformational state due to simultaneously binding of structurally distinct vRNA panhandle and single strand RNA substrates. Although the role of dual RNA binding modes in the virus replication cycle is unknown, their involvement in the packaging of viral genome and regulation of CCHFV replication in conjunction with RdRp and host derived RNA regulators is highly likely.

## Introduction

Crimean-Congo hemorrhagic fever virus (CCHFV) is a tick-borne nairovirus within the *Bunyaviridae* family, which causes severe hemorrhagic fever with a mortality of 30% in more than thirty countries worldwide [1–4]. Lack of anti-CCHFV vaccine and limited therapeutic interventions have raised concerns about this zoonotic illness. This virus is transmitted to humans by either tick bites or direct contact with contaminated blood or tissue samples from the infected hosts [5,6]. The viral genome is composed of three negative sense RNA segments (S, M and L), which encode nucleocapsid protein (N protein), glycoprotein precursor (GPC), and RNA-dependent RNA polymerase (RdRp), respectively [7]. The hallmark of the *Bunyaviridae* genomic RNA is the partially complementary nucleotide sequence at the 5’ and 3’ termini that have been reported to undergo intramolecular hydrogen bonding and form the panhandle structure [8–11]. N protein remains associated with the viral genome and forms three nucleocapsids that serve as templates for the synthesis of viral mRNA and replication of the viral genome. CCHFV and hantavirus N protein, both members of the *Bunyaviridae* family, interact with RdRp. The interaction plays a role in virus replication [12,13]. The complementary RNA (cRNA), an intermediate for the replication of the viral genome also forms nucleocapsid in association with the N-protein. In comparison, the viral mRNA is naked and does not form nucleocapsids. The encapsidation of viral genome is also required for the packaging of nucleocapsids into new virions. Interestingly, the nucleocapsids generated from cRNA are not packaged into progeny virions. The assembly process in bunyaviruses is generally mediated by direct association between nucleocapsids and viral glycoproteins [14–16]. It still remains unclear how N protein selectively recognizes viral genomic RNA for the selective encapsidation and packaging into progeny virions. The viral genome must harbor packaging signals that are specifically recognized by N protein for selective encapsidation and packaging. However, such packaging signals have not yet been identified for CCHFV. To this end, we studied the interaction of bacterially expressed and purified CCHFV N protein with the synthetic S-segment viral RNA (vRNA). We report that CCHFV N protein has distinct binding modes for double strand RNA (dsRNA) and single strand RNA (ssRNA). N protein specifically recognizes the viral RNA panhandle through the dsRNA binding mode. However, N protein does not discriminate between viral and nonviral RNAs through ssRNA binding mode. N protein undergoes a conformational change after binding to the substrate ssRNA or dsRNA. In addition, N protein can simultaneously bind to both the panhandle and ssRNA, and undergoes a unique conformational change due to simultaneous binding. Although the role of dual RNA binding modes in virus replication remains unknown at present, their role in the regulation of virus replication in conjunction with RdRp and host factors can’t be ruled out (explained in the discussion). Recently, the X-ray crystallographic structure of CCHFV N protein at 2.3 Å resolution has demonstrated a unique metal based endonuclease activity, which also provided insights into RNA binding and oligomerization of N protein [17–19]. Apart from packaging the viral genome, the bunyaviridae N protein has been reported to play diverse roles in the virus replication cycle [20–24].

## Materials and methods

### Reagents

PCR primers were from Invitrogen. All other PCR reagents were from New England Biolabs and Genscript. The radioactive [α^32^P] CTP was from Perkin Elmer. T7 transcription and RNA purification kits were from Promega. RNeasy and other RNA purification kits were from Qiagen. NiNTA beads were from Gold Bio. All other chemicals were purchased from Sigma.

### Cloning, expression and purification of CCHFV N protein

The plasmid expressing the CCHFV N protein (strain 10200) was gifted by Stuart T Nichol from the National Center for Emerging and Zoonotic Infectious Diseases, Centers for Disease Control and Prevention (CDC), Atlanta, Georgia. The N protein ORF was PCR amplified using two opposing primers and cloned between NdeI and XhoI restriction sites in pET30a backbone. The resulting plasmid (pET-CCHFNP) expressing the N protein as C-terminally His-tagged fusion protein was transformed into *Escherichia coli* Rosetta (DE3) cells (Novagen). To induce the protein expression, isopropyl-D-1-thiogalactopyranoside (IPTG) was added to the bacterial culture at a final concentration of 0.5mM, when the optical density of bacterial culture at 600 nm reached 0.6. The culture was grown for additional 20 hours at 16°C. Cells were harvested by centrifugation and resuspended in lysis buffer (50 mM Tris-HCl, pH 7.4, 150 mM NaCl, 2 mM dithiothreitol [DTT], 1% Triton-X100, 5 mM CHAPS, 0.1 mM phenyl methyl sulfonyl fluoride [PMSF]). The resuspended cells were sonicated on ice and cleared by centrifugation. The cleared cell lysate was loaded onto a NiNTA column (Gold Bio), pre equilibrated with the lysis buffer. The column was then washed twice with lysis buffer, followed by an additional wash with wash buffer (50 mM Tris, 500 mM NaCl, 0.05% Triton X-100 and 20mM Imidazole). The bound N protein was eluted with the elution buffer (50 mM Tris, 500 mM NaCl, 0.05% Triton X-100 and 250mM Imidazole). The eluted protein was quantified and stored in 100 μl aliquots at -80°C. The Sin Nombre hantavirus nucleocapsid protein was purified as C-terminally His tagged fusion protein, as previously reported [25].

### T7 transcription for RNA synthesis

**Fig 1:**
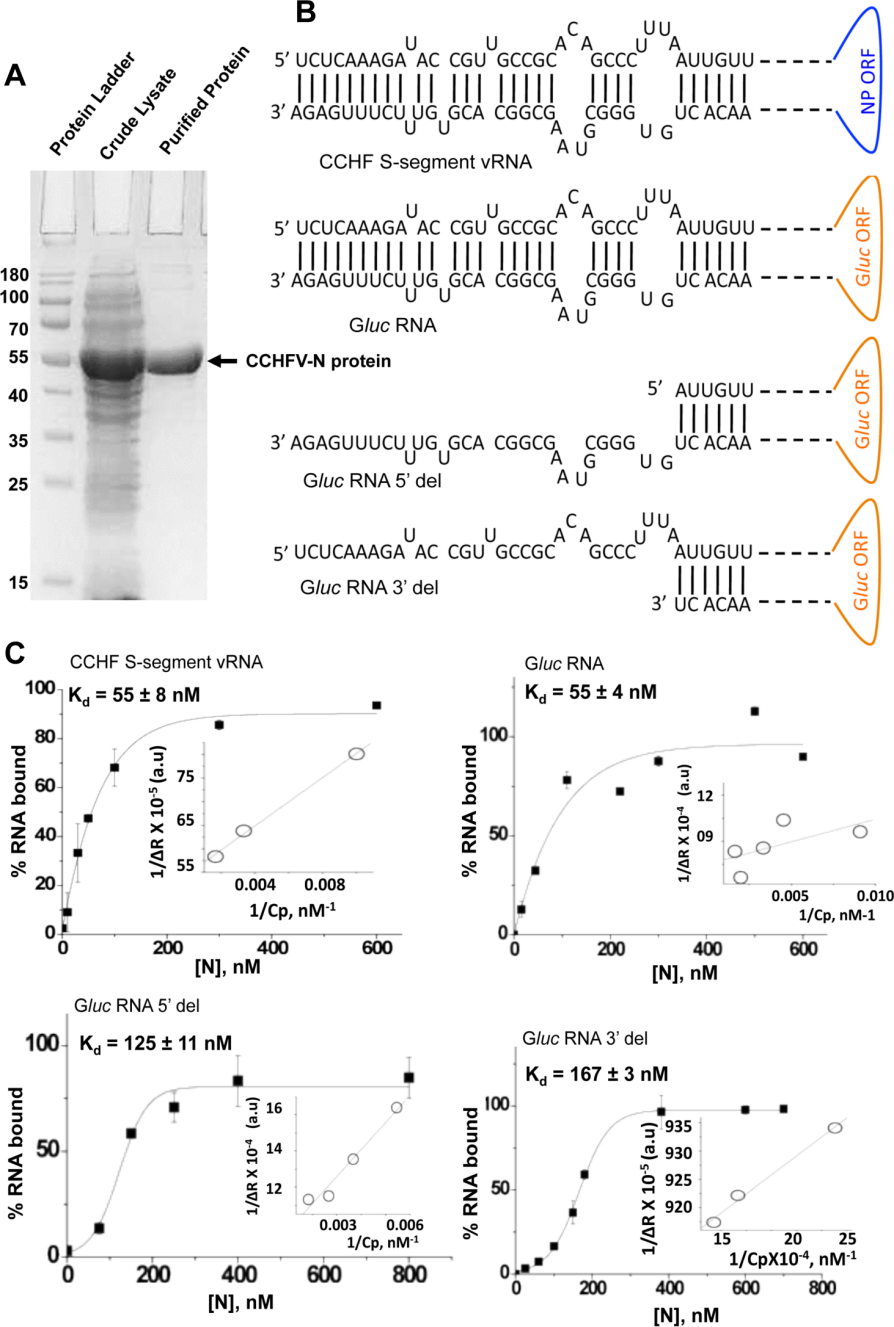
Filter binding analysis. (A). SDS-PAGE of bacterially expressed and purified CCHFV N protein. Lane 1 shows the protein ladder. Lane 2 shows the bacterial lysate before the purification of N protein. Lane 3 shows the CCHFV N protein purified by NiNTA column chromatography using native purification protocol. (B). The RNA molecules shown in this panel were synthesized by T7 transcription and tested for binding with purified N protein. The prediction of secondary structures by mFold revealed that complementary nucleotides from 5’ and 3’ termini undergo base pairing and form hairpin structure, referred as panhandle structure in this manuscript. The nucleotides deleted at the 5’ terminus (G*luc* RNA 5’ del) and 3’ terminus (G*luc* RNA 3’ del) are not shown. (C). Binding profiles for the purified CCHFV N protein with the RNA molecules from panel B are shown. Increasing concentrations of N protein were added to a fixed concentration of [α^32^P] CTP labeled RNA and the mixture was incubated at room temperature for 45 minutes, followed by filtration through nitrocellulose filter. The RNA-N protein complex retained on the filter was quantified by scintillation counter and the radioactive signal was used to calculate the percent RNA bound at each input concentration of N protein using Eq 1, to generate the binding profile. Inset shows the double reciprocal plot for the calculation of ΔR_max_, used to calculate the percent bound RNA in Eq 1. The dissociation constant (K_d_) was calculated as mentioned in materials and methods. Standard deviation was calculated from three independent experiments.

**Fig 2:**
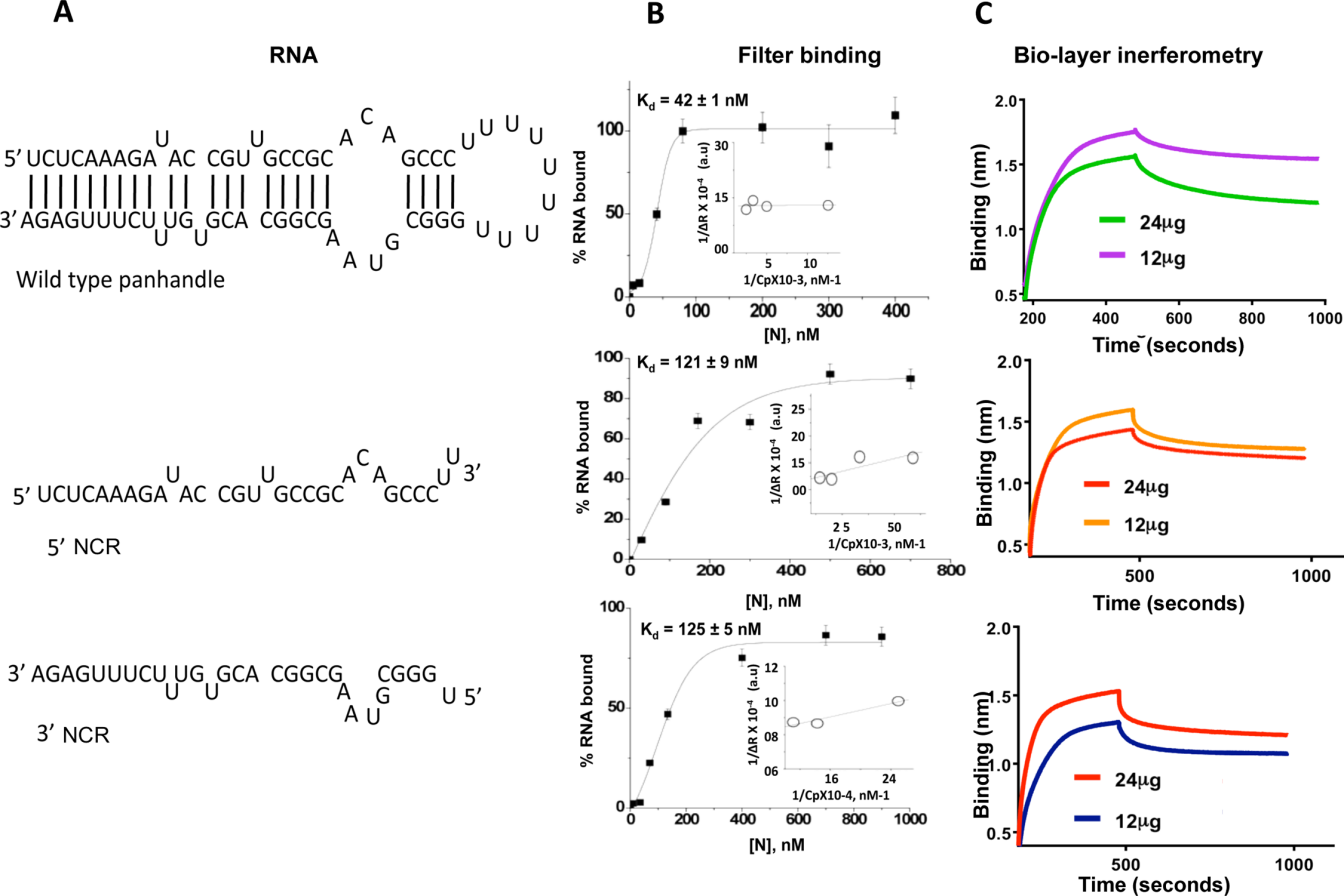
Comparison of binding affinities by filter binding analysis and biolayer interferometry. (A). Sequence and most probable secondary structure of wild type panhandle from S-segment vRNA. The thirty nucleotides from 5’ and 3’ termini of S-segment vRNA panhandle are shown below the wild type panhandle. (B). Interaction of purified N protein with the [α^32^P] CTP labeled RNA molecules shown in panel A was carried out by filter binding analysis, as mentioned in Fig 1 and also in materials and methods. The binding profiles from filter binding analysis are shown next to the respective RNA. Insets show the double reciprocal plots as mentioned in Fig 1C. (C). Binding analysis of purified N protein with RNA molecules shown in panel A was also carried out using Bio-layer interferometry (BLI). Representative BLI sensograms showing over time association and dissociation of N protein (0.22 μM or 0.44 μM) with the RNA of interest. The BLI sensograms are shown next to the representative RNA.

**Fig 3:**
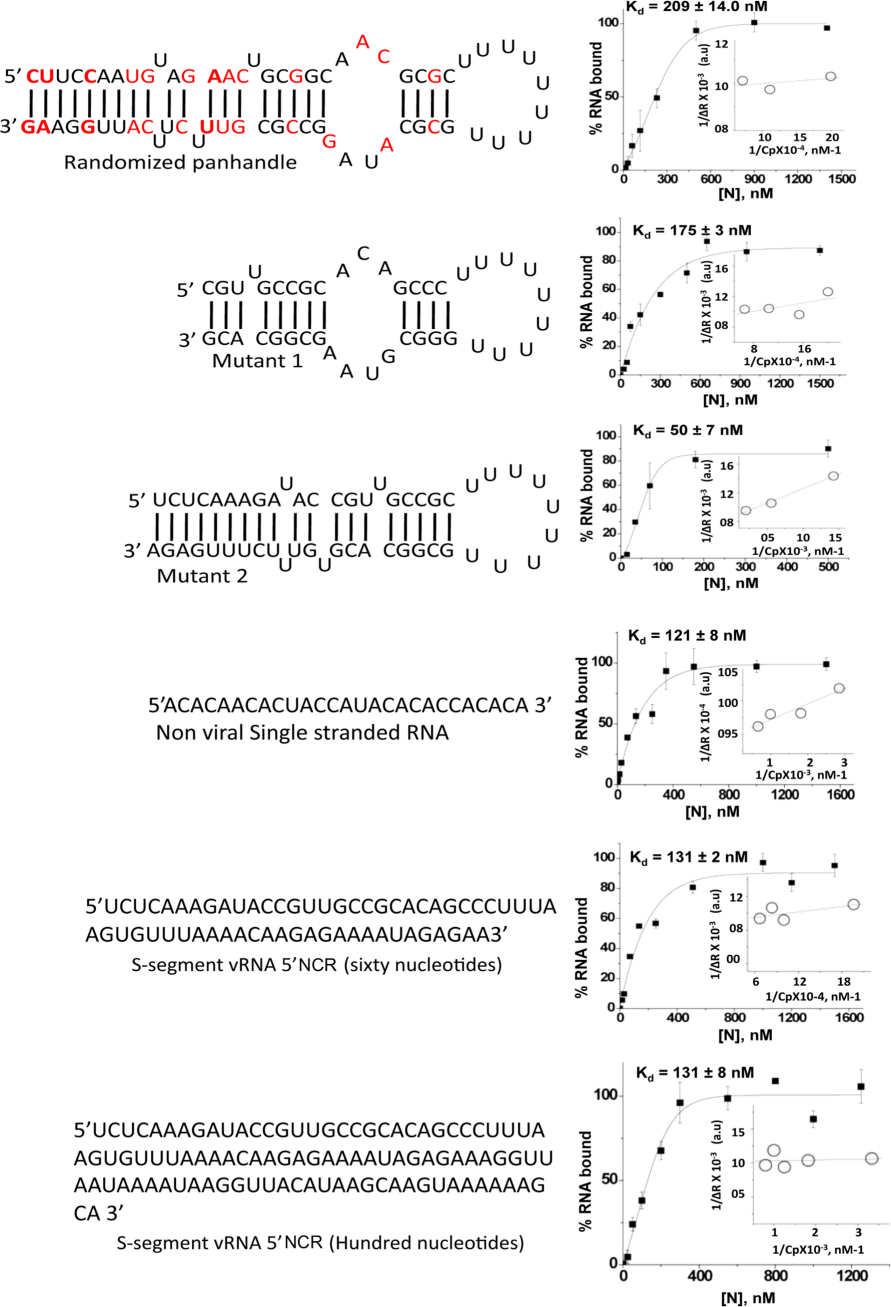
RNA filter binding analysis. Sequence and most probable secondary structure of randomized panhandle, mutants 1 and 2 of wild type CCHFV S-segment vRNA panhandle are shown on the left. The nonviral single strand RNA molecule does not have a secondary structure. The sixty and hundred nucleotide long 5’ NCR sequences of CCHFV S-segment vRNA have very weak secondary structure (ΔG~6.0 Kcal/mol), and likely remain single stranded. All six RNA molecules in the left were synthesized by T7 transcription and radiolabeled by [α^32^P] CTP. Interaction of purified N protein with radiolabeled RNA molecules was carried out by filter binding analysis as mentioned in Fig 1 and also in materials and methods. The respective filter binding profiles corresponding to each RNA are shown on the right. The binding reactions were carried out in RNA binding buffer at 250 mM NaCl. Insets show the double reciprocal plots as mentioned in Fig 1C.

We used a fusion PCR strategy to amplify the DNA sequence encoding the CCHFV S-segment vRNA. Briefly, a forward primer containing a flanking T7 promoter and a reverse primer was used to PCR amplify the 5’ noncoding region (NCR) of the S-segment gene, along with downstream twenty nucleotides from the N protein ORF. Similarly a pair of primers was used to PCR amplify the region encoding the N protein ORF along with twenty nucleotides from both 5’ and 3’ NCR. Same strategy was used to PCR amplify the 3’ NCR along with twenty nucleotides upstream of the 3’ NCR from the N protein ORF. The three PCR products containing overlapping regions were gel purified, mixed together, and used as template along with two terminal primers to generate the final PCR product containing the full length S-segment gene with a flanking T7 promoter. The PCR product was used in a T7 transcription reaction for the synthesis of CCHFV S-segment vRNA, as previously reported [12,22,26–28]. We used a mini genome plasmid containing gaussia luciferase in opposite orientation, flanked by 5’ and 3’ NCR sequences of CCHFV S-segment vRNA, as template along with a forward primer containing a flanking T7 promoter and a reverse primer to generate the PCR product. The mini genome plasmid was a gift from Stuart T Nichole (CDC). The PCR product was gel purified and used as template in a T7 transcription reaction for the synthesis of G*luc* RNA in Fig 1. The purified PCR product was then used as template along with appropriate primers to generate truncated PCR products for the synthesis of G*luc* RNA 5’ del and G*luc* RNA 3’ del in Fig 1. For the synthesis of shorter RNA molecule including wild type and mutant panhandles used in Figs 2 and 3, two complementary DNA oligos having appropriately positioned T7 promoter were annealed together. The annealed oligoes were subjected to ten rounds of PCR amplification to fill the incomplete ends, if any. The resulting PCR products were gel purified and used in T7 transcription reaction for the synthesis of RNA of interest. The RNA molecules were radiolabeled during synthesis using [α^32^P] CTP as previously reported [12,22,26–28]. The DNA templates were digested using DNAse1. The radiolabeled RNA was purified using trizol reagent and RNeasy purification kits (Qiagen), as previously reported [12,22,26–28]. As required, the RNA was further purified by gel extraction using denaturing PAGE, as previously reported [23].

### RNA filter binding analysis

Interaction of bacterially expressed and purified CCHFV N protein with the RNA of interest (Figs 1, 2 and 3 and Table 1) was studied by filter binding assay, as previously reported [29]. Briefly, the desired RNA molecules were synthesized *in vitro* using T7 RNA polymerase and radiolabeled with [α^32^P] CTP during synthesis, as mentioned above. All binding reactions were carried out in RNA binding buffer (40 mM Tris-HCl [pH 7.4], 80 mM NaCl, 20 mM KCl, 1 mM dithiothreitol) at a constant concentration of RNA with increasing concentrations of N protein. Reaction mixtures were incubated at room temperature for 30–45 min and filtered through nitrocellulose membranes under vacuum. Filters were washed with 10 ml of RNA binding buffer and dried. The amount of RNA retained on the filter at each input concentration of N protein was measured by quantifying the radioactive signal, using a scintillation counter. The background signal from nonspecific binding of RNA to the filter was subtracted from each data point. A binding profile was generated by plotting the radioactive signal along Y-axis and N protein concentration along X-axis. The percentage of bound RNA at each input concentration of N protein was calculated using the Eq 1.

**Table 1:** Binding parameters calculated by filter binding analysis for the association of CCHFV N protein with different RNA molecules in RNA binding buffer at two NaCl concentrations.

Name of RNA	K_d_ (nM)	
	NaCl (80 mM)	NaCl (250 mM)
**CCHF S-segment vRNA**	55 ± 8	55 ± 8
**G*luc* RNA**	55 ± 4	43 ± 4
**G*luc* RNA 5’ del**	125 ± 11	134 ± 5
**G*luc* RNA 3’ del**	167 ± 3	150 ± 5
**Wild type panhandle**	42 ± 1	47 ± 2
**5’ NCR**	121 ± 9	147 ± 9
**3’ NCR**	125 ± 5	137 ± 11

Note: All binding reactions were carried out at room temperature.

$$\%\;bound\;RNA=\Delta R\;/\;\Delta R_{max}*100$$ (Eq 1)

Where ΔR is the change in radioactive signal at each addition of N protein. ΔR_max_ is the same parameter when the RNA is totally bound to the N protein. Double reciprocal plot (1/*ΔR* versus 1*/C*_*p*,_ [29]) was used to calculate the value of *ΔR*_*max*_, using Eq 2. C_p_ is the input protein concentration.

$$1/\Delta R=1/\Delta R_{max}+K_{d}/\;(\Delta R_{max}*C_{p}).$$ (Eq 2)

Since Eq 2 is valid for the calculation of *ΔR*_*max*_ under the conditions where Cp >> initial concentration of RNA [30], only the data points corresponding to the saturation phase of the binding profile were fitted to the Eq 2 for the calculation of *ΔR*_*max*_. Alternatively, the *ΔR*_*max*_ was calculated by simply averaging the radioactive signal of saturating data points. The percentage of bound RNA obtained from Eq 1 was plotted verses input N protein concentration, and the resulting data points were fit to a dose-response equation using the program Origin 6 (Microcal). The apparent dissociation constant (*Kd*) corresponded to the concentration of N protein required to obtain the half-saturation in the fitted binding curve, assuming that the complex formation obeys a simple bimolecular equilibrium.

The binding profiles showing sigmoidal behavior were examined for cooperativity using the Hill equation (Eq 3) [31,32]. A plot of ΔR/ΔR_max_ verses input N protein concentration was fit to the Eq 3.

$$\phi ={\left[X\right]}^{n}/\;K_{d}+{\left[X\right]}^{n},$$ (Eq 3)

where Φ (ΔR / ΔR_max_) is the fraction of RNA bound at each input concentration of N protein, [X] denotes the input concentration of N protein, K_d_ is the dissociation constant and “n” represents the Hill coefficient. If n < 1 the system exhibits the negative cooperativity, if N > 1 the system exhibits the positive cooperativity, if n = 1, the system does not have the cooperativity.

### RNA secondary structure analysis

We used mFold to analyze the most probable secondary structures of RNA molecules shown in Figs 1–3, as previously reported [33]. While examining the suboptimal secondary structures predicted by mFold, we determined the P-num values for each of the nucleotides in the RNA. The P-num value of a particular nucleotide in the RNA sequence represents the number of potentially stable pairing partners for that nucleotide elsewhere in the same RNA molecule. Viewing the P-num values from the context of suboptimal folds, the structures composed of low P-num nucleotides still form and are composed of bases with the fewest alternative pairing partners [34].

### RNA competition experiments

Competition experiments were performed using filter binding assay as previously reported [33]. A constant concentration of bacterially expressed and purified CCHFV N protein was incubated with a constant concentration of [α^32^P] CTP labeled RNA and increasing concentrations of cold competitor RNA of interest. The reaction mixture was incubated at room temperature for 45 min and then filtered through nitrocellulose filters under vacuum. The bound radiolabeled RNA retained on the filter at each input concentration of the competitor RNA was measured using scintillatation counter. Percent radiolabeled RNA bound to the protein was plotted against input competitor RNA concentration.

### Fluorescence binding

The binding of wild type vRNA panhandle, 5’ and 3’ NCR sequences with CCHFV N protein was carried out in Shimadzu spectrofluorometer RF-5301PC as previously reported [28,29]. Fluorescence spectrum of N protein from 300–500 nm was recorded in RNA binding buffer using an excitation wavelength of 295 nm (excitation slit width, 5 nm; and emission slit width, 10 nm). The unaltered fluorescence signal of N protein over time at 334 nm indicated the absence of photodegradation. All fluorescence binding experiments were carried out at room temperature. To a fixed concentration of N protein, the RNA of interest was added at increasing concentrations, and the fluorescence spectrum of N protein at 300–500 nm was recorded at each input concentration of the RNA. To calculate the dissociation constant (*Kd*), the fluorescence intensity value at 334 nm at each input concentration of the ligand was recorded. The binding profile was generated by plotting the fluorescence value at 334 nm along the Y axis and ligand concentration along the X axis. The percentage of N protein bound to the RNA of interest at each input concentration of the RNA was calculated from Eq 4.

$$\%bound=\Delta F/\Delta F_{max}*100.$$ (Eq 4)

Where ΔF is the change in fluorescence signal at 334 nm at each addition of RNA. ΔF_max_ is the same parameter when N protein is totally bound to the RNA. Double reciprocal plot (1/*ΔF* versus 1*/C*_*p*,_ [29]) was used to calculate the value of *ΔF*_*max*_, using Eq 2. C_p_ is the input RNA concentration.

$$1/\Delta F=1/\Delta F_{max}+K_{d}/(\Delta F_{max}*C_{p}).$$ (Eq 5)

Since Eq 5 for the calculation of *ΔF*_*max*_ is valid under the conditions where Cp>> initial concentration of N protein, only the saturating data points of the binding profile were fitted to Eq 4 for the calculation of *ΔF*_*max*._ A plot of percent bound N protein versus RNA concentration was used for the calculation of apparent dissociation constant *(K*_*d*_*)*, which corresponds to the concentration of RNA required to obtain half saturation, assuming that complex formation obeys a simple bimolecular equilibrium.

### Stern volmer quenching analysis

To monitor the conformational changes in N protein due to binding with the RNA of interest, we monitored the quenching of tryptophan residues of N protein by a neutral quencher (acrylamide) before and after the binding of RNA of interest, according to the Stern-Volmer equation [35] *F*_0_/*F* = 1 + *K*sv[*Q*], where *F*_0_ and *F* are the fluorescence intensities in the absence and presence of the quencher, respectively, [*Q*] is the concentration of the quencher, and *K*sv is the Stern-Volmer quenching constant. Small aliquots (1–5 μl) of acrylamide from concentrated stock solutions (3M-10M) were added to 300 μl of N protein at a concentration of 250 nM in the binding buffer, and the tryptophan fluorescence signal at 334 nm was recorded at each input concentration of acrylamide. The total volume of added acrylamide solution was less that 10% the total volume of N protein solution in the cuvette. Similar quenching studies were carried out with N protein that was pre-incubated with the desired concentration of RNA of interest. The data points in a plot of *F*_0_/*F versus* [*Q*] fit to a single straight line, except when N protein was simultaneously bound to both the vRNA panhandle and single strand 3’ or 5’ NCR. Fitting of data points to a single straight line indicates a single species of tryptophan residues that remain equally accessible to the neutral quencher. The Stern-Volmer quenching constant (*K*sv) was calculated from the slope of the straight line fitted to the data points as reported previously [26,33].

### bis-ANS binding

Fluorescence studies of hydrophobic fluorophore bis-ANS were carried out in Shimadzu spectrofluorometer (RF-5301PC), as previously reported [26]. The fluorophore was dissolved in dimethyl sulfoxide, and its concentration was determined from the extinction coefficient (ϵ396 = 24,000 M−1 cm−1). The fluorophore was excited at 399 nm, and fluorescence emission was recorded at 485 nm. Small aliquots of bis-ANS were added from a higher concentration stock to a fixed concentration of either N protein alone or N protein in the presence of RNA of interest, and the fluorescence value at 485 nm was recorded at each input concentration of bis-ANS. To determine the change in fluorescence signal of bis-ANS due to binding with N protein or N protein-RNA complex, the fluorescence signal of free bis-ANS in binding buffer was subtracted.

### Biolayer interferometry

Biolayer interferometry was used to measure the binding affinities of N protein with RNA of interest using the BLItz system (ForteBio Inc.), as previously reported [28]. Briefly, the Biotinylated RNA was synthesized and loaded onto Streptavidin biosensors (catalog no. 18–5019, Forte Bio Inc.). After mounting, biosensors were equilibrated with RNA binding buffer and then dipped in purified N protein preparation at concentrations 12 and 24 μg/ml for the measurement of association and dissociation kinetics. The settings were as follows: initial base line for 30 s, loading for 120 s, base line for 30 s, association for 300s, and dissociation for 500 s. The kinetic parameters K_ass_ (association rate constant), K_dis_ (dissociation rate constant) and the binding affinity (K_d_ = K_dis_/K_ass_) were calculated with the help of data analysis software (BLItZ Pro). All the experiments were performed at room temperature.

### *In slico* analysis

The 3D structure of CCHFV N protein from the strain-10200 (Accession number U88410) was predicted using the known X-ray crystal structure of similar proteins including CCHFV N protein (strain YL04057) as template. The 3D structure was generated using I-TASSER (http://zhanglab.ccmb.med.umich.edu/I-TASSER), a web based server. The electrostatic surface potential of N protein was generated using PyMol viewer software tool, and the Potential RNA-binding region of CCHFV N protein was predicted and labeled, based on the surface positively charged grooves and previously reported similar analysis for the CCHFV N protein from the strain YL04057 [36].

### RNA helicase assay

To determine whether N protein unwinds the double strand RNA after binding, we performed RNA helicase assay as previously reported [37]. Briefly, as shown in Fig 2A the 5’ and 3’ termini of CCHFV S-segment vRNA are partially complementary and undergo base pairing to form a panhandle structure. We synthesized separately thirty nucleotides from 5’ and 3’ termini of S-segment vRNA by *in vitro* T7 transcription reaction, containing a stretch of uracil residues. The 3’ sequence was radiolabeled by α-P32-GTP during synthesis. The radiolabeled 3’ sequence and unlabeled 5’ sequence was mixed in 1:1 ratio in buffer A (40 mM HEPES pH 7.4, 80 mM NaCl, 20 mM KCl, and 1 mM DTT), heated at 95°C for 3 min and incubated at RT for 3 h. The hybridized RNA was gel-purified and used as substrates in the helix destabilization reactions, as previously reported ^*(36*^. Twenty microliter reactions containing 100 nM N protein of either CCHFV or SNV and 15 nM RNA substrate in the binding buffer containing 1 mM Mg^2+^ and 1 mM ATP were incubated at 37°C for different time intervals and terminated by the addition of 4 μL of RNA sample buffer (100 mM Tris HCl at pH 7.4, 50 mM EDTA, 0.1% triton X-100, 0.5% SDS, 50% glycerol, and 0.1% bromophenol blue). The products were fractionated on 10% SDS PAGE. Gels were exposed to PhosphorImager screens for ~18 hours and analyzed. Helix destabilization activity was monitored by the release of radiolabed 3’ sequence from the double strand panhandle.

## Results

### Interaction of CCHFV N protein with viral S-segment RNA

As discussed in materials and methods the CCHFV N protein from the strain 10200 (NCBI accession number U88410.1) was expressed in *E*.*coli* as a N-terminal His-tagged fusion protein. The protein was purified by NiNTA chromatography using a native purification procedure. As shown in Fig 1A, the purified N protein was free of detectable heterologues bacterial proteins and its electrophoretic mobility was consistent with its expected molecular mass (54 KDa). The viruses of the *Bunyaviridae* family harbor noncoding regions (NCR) at both the 5’ and 3’ termini of the viral genome. Similar to other members of the *Bunyaviridae* family, the 5’ and 3’ NCR sequences of the CCHFV genome are partially complementary and likely undergo base pairing to form the terminal panhandle structure. Depending upon the sequence, length and complexity, an RNA molecule can potentially form multiple secondary structures. However, as mentioned in Materials and Methods, the p-num analysis of CCHFV S-segment RNA revealed that p-num values for the 5’ and 3’ nucleotides is either 1 or 0, where paired nucleotides have a value of 1 and unpaired nucleotides have a value of 0. This strongly supports that pairing partners for 5’ nucleotides are located at the 3’ terminus of the genome, resulting in the formation of a panhandle structure as shown in Fig 1B. Formation of such panhandle structures have been previously reported for other Bunyaviruses [8–11]. Similar analysis revealed the formation of panhandle structure by the 5’ and 3’ NCR sequences fused to the G*luc* RNA (Fig 1B).

To determine whether CCHFV genome harbors cis-acting structural motifs that might be specifically recognized by the CCHFV N protein, we studied the interaction of purified N protein with the synthetic CCHFV S-segment RNA using filter binding analysis, as mentioned in materials and methods. The RNA was synthesized *in vitro* by T7 transcription reaction. The filter binding analysis revealed that N protein bound to the S-segment vRNA with a dissociation constant (K_d_) of 55 ± 8 nM (Fig 1C). We did not observe a noticeable change in the binding affinity at high salt concentration (Table 1). We next synthesized the G*luc* RNA by *in vitro* T7 transcription reaction (Fig 1B), as mentioned in materials and methods. The G*luc* RNA has Gaussia luciferase open reading frame (G*luc* ORF) in opposite orientation flanked by 5’ and 3’ NCR sequences of CCHFV S-segment vRNA. The filter binding analysis revealed that N protein bound to the G*luc* RNA with similar affinity as CCHFV S-segment vRNA (Fig 1C). Again the binding affinity was not altered by high salt concentration (Table 1). This initial experiment suggested that region of S-segment vRNA encoding the N protein does not play a role in binding with the N protein. It also suggests that high affinity-binding site for N protein may be located in the vRNA termini. Deletion of 32 and 31 nucleotides from either 5’ and 3’ termini, respectively, of G*luc* RNA resulted in almost three-fold decrease in binding affinity with N protein (Fig 1C and Table 1), further supporting the location of high affinity binding site at the termini of G*luc* RNA. Again the K_d_ values with these truncated RNAs were insensitive to high salt concentrations (Table 1). We also studied the interaction of N protein with the 30 nucleotide long homopolmer poly (U). Interestingly, the poly (U) RNA showed a very weak binding with the N protein and K_d_ value could not be calculated. Other investigators also reported the weak binding between N protein and poly (U) RNA [36]. Taken together, these observations suggest that vRNA termini are likely required for high affinity binding to the N protein.

To further confirm the results of the Fig 1, The radiolabeled RNA molecules synthesized by T7 transcription were purified by trizol reagent, followed by further purification by gel extraction using denaturing PAGE. The gel purified RNA molecules were homogenous in size and were retested for N protein binding using filter binding assay (S1 Fig). We did not observe a noticeable change in the binding parameters after further purification using the gel extraction approach (compare binding parameters from Fig 1C with S1 Fig). Since K_app_(1/K_d_) = K_0_[n], where K_app_ is the apparent binding constant, K_0_ is the intrinsic binding constant and n is the binding stoichiometry, a possible difference in reported binding affinities due to potential variation in binding stoichiometries cannot be ruled out. It is evident from Fig 1C that binding profiles with G*luc* RNA 5’ del and G*luc* RNA 3’ del show a sigmoid behavior. This sigmoidal behavior was also observed with gel purified G*luc* RNA 3’ del (S1 Fig). These binding profiles were fit to Hill equation, which revealed a Hill coefficient of greater than 1.0 (not shown), suggesting a positive cooperativity in the binding process, which is further explained in the discussion.

### The terminal panhandle is sufficient for high affinity binding with the N protein

We next synthesized a wild type panhandle RNA composed of 30 nucleotides from both 5’ and 3’ termini of CCHFV S-segment vRNA, separated by a six nucleotide long uracil loop (Fig 2A). In addition, the thirty nucleotides from 5’ and 3’ termini were separately synthesized by T7 transcription and radiolabelled during synthesis. The mFold analysis revealed that 30 nucleotide long NCR sequences from 5’ and 3’ termini do not form stable secondary structures and mostly remain single stranded. Filter binding analysis was carried out to study the interaction of these shorter RNA segments with the purified N protein. As shown in Fig 2B, N protein bound to the wild type panhandle with a K_d_ value of 42±1 nM. The binding affinity was insensitive to high salt concentration (Table 1). It is evident from Table 1 that N protein binds to both the wild type panhandle and full length S-segment vRNA with similar affinity, suggesting that terminal panhandle is sufficient for high affinity binding to the N protein. The short 5’ and 3’ NCR sequences bound to N protein with the K_d_ values of 121±9 nM and 125±5 nM, respectively, an affinity three fold lower compared to the wild type panhandle (Fig 2B). Again the binding affinity was not significantly affected by high salt concentration (Table 1). To confirm the results from filter binding analysis, we examined the association and dissociation kinetics of vRNA panhandle, 5’ and 3’ NCR with purified N protein, using Biolayer Interferometry (Fig 2C), as previously reported [28]. The association kinetics appears to have a biphasic behavior while the dissociation kinetics appears monophasic in nature (Fig 2C). The appearance of biphasic behavior could be due to RNA induced conformational changes in N protein. It is evident from Fig 2 and Table 2 that N protein bound to vRNA panhandle with higher affinity as compared to 5’ and 3’ NCR, further confirming the results from filter binding assay. Comparing the results from Fig 2 with the Fig 1, it is clear the N protein binds to short 5’ and 3’ NCR sequences with similar affinity as G*luc* RNA having the deletion of thirty nucleotides at either 3’ or 5’ terminus (Table 1). Collectively, the results from Figs 1 and 2 demonstrate that N protein binds with higher affinity to the structured vRNA panhandle in comparison to unstructured 5’ and 3’ NCR sequences.

**Table 2:** Binding parameters calculated by Biolayer interferometry for the association of CCHFV N protein with different RNA molecules in RNA binding buffer containing 80 mM NaCl.

RNA	K_d_ (nM)	K_ass_ (M^-1^s^-1^)	K_diss_ (s^-1^)
**Wild type panhandle**	41 ± 3	2.15x10^4^	8.93x10^-4^
**5’ NCR**	131 ± 3	1.35x10^4^	1.77x10^-3^
**3’ NCR**	124 ± 2	1.37x10^4^	1.71x10^-3^

Note: All binding reactions were carried out at room temperature.

To further confirm the results of Fig 2, the radiolabeled RNA molecules were further gel purified using denaturing PAGE. The gel purified RNA molecules were homogenous in size (S2 Fig). The binding analysis of gel purified RNA molecules with N protein did not show a noticeable change in the binding parameters (compare binding parameters from Fig 2 with S2 Fig).

### N protein binding to vRNA panhandle is sequence specific

To determine whether N protein binding to the wild type panhandle is sequence specific, we randomized the primary sequence of the wild type panhandle but retained the secondary structure. The filter binding analysis predicted that N protein bound to randomized panhandle with significantly weaker affinity compared to wild type panhandle, suggesting that primary sequence of the wild type panhandle is required for high affinity binding (Fig 3). To further characterize the binding with the vRNA panhandle, we deleted 12 and 13 nucleotides from 5’ and 3’ terminus of wild type panhandle, respectively (mutant 1). Interestingly, this terminal deletion significantly impacted the binding with N protein. In comparison, the deletion of 9 nucleotides from both 5’ and 3’ termini close to the uracil loop (mutant 2) did not affect the binding (Fig 3). This observation clearly demonstrates that terminal nucleotides of the panhandle are required for high affinity binding with the N protein. To determine whether the binding with the unstructured RNA was also sequence specific, we synthesized a thirty nucleotide long non-viral RNA. Interestingly, the filter binding analysis revealed that N proteins bound to this nonviral single strand RNA with similar affinity as unstructured 30 nucleotide NCR sequences from 5’ and 3’ termini of CCHFV S-segment vRNA.

Since viral genome of negative strand RNA viruses is cotranscriptionally encapsidated by the nucleocapsid protein, it is likely that 5’ terminus of the viral genome becomes first available for encapsidation during virus replication. We synthesized sixty and hundred nucleotide long 5’ terminal sequences of CCHFV S-segment vRNA. The RNA molecules were radiolabeled during synthesis, purified by trizol reagent, followed by further purification by gel extraction using denaturing PAGE (S3A Fig, lanes 5 and 6). An examination by filter binding analysis revealed that both the RNA molecules bound to N protein with a K_d_ value of ~131 nM (Fig 3). Similar binding affinity was observed with thirty nucleotide long 5’ NCR sequence of S-segment vRNA (Fig 2 and S2 Fig). To further confirm the results of Fig 3, the remaining RNA molecules were gel purified and retested for N protein binding using filter binding assay. We observed a similar trend in binding parameters after the gel purification of RNA molecules (compare Fig 3 with S3B Fig).

### N protein has distinct binding modes for vRNA panhandle and single strand RNA

**Fig 4:**
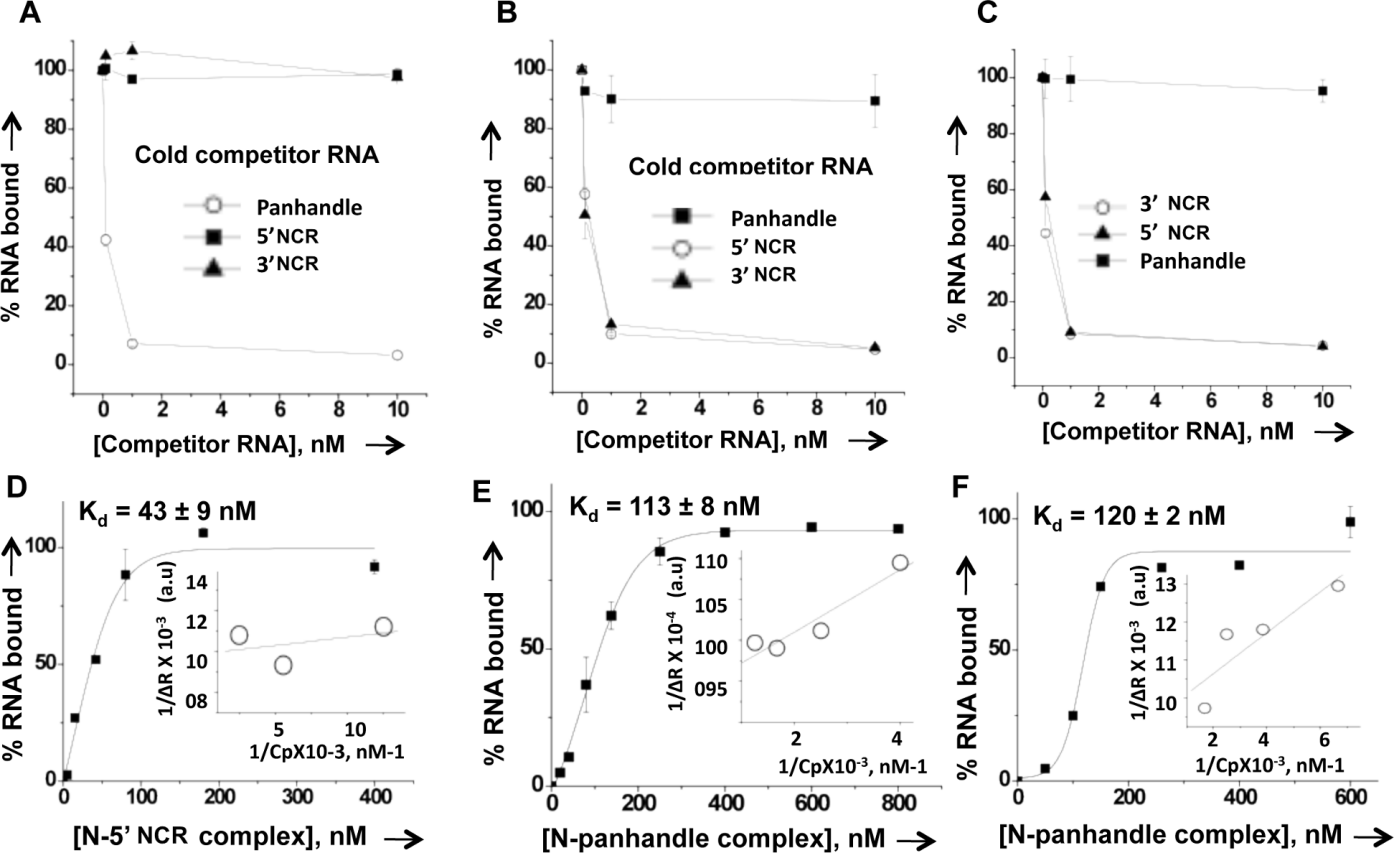
RNA binding competition experiment. (A). As previously reported [33], a fixed concentration of CCHFV N protein (200 nM) was incubated with a constant concentration of [α^32^P] CTP labeled wild type CCHFV S-segment vRNA panhandle (0.01nM) and increasing concentrations (0.1 nM, 1 nM, and 10 nM) of competitor cold vRNA panhandle (open circle), thirty-nucleotide long 5’ NCR sequence (filled square) and thirty nucleotide long 3’ NCR sequence (filled triangle). (B and C). The competition experiment performed in these panels was carried out similar to panel A, except CCHV V N protein (350 nM) was incubated with the constant concentration (0.01nM) of P^32^-CTP labeled 5’ NCR (panel B) or P^32^-CTP labeled 3’ NCR (Panel C) and increasing concentrations of competitor RNA as mentioned in panel A. (D). To determine whether N protein can simultaneously bind both vRNA panhandle and 5’ UTR sequence, a filter binding assay was performed in which a fixed concentration of radiolabeled vRNA panhandle was incubated with increasing concentrations of N protein-5’ NCR complex. The complex was generated by incubating N protein with saturating concentrations of cold 5’ NCR sequence, as mentioned in the text. Reaction mixtures were filtered through a nitrocellulose filter, and the percentage of hot S segment RNA retained on the filter was plotted *versus* input concentrations N-5’ NCR complex to generate the binding profile for the calculation of *Kd*. In a similar experiment increasing concentrations of preformed complex between N protein and cold vRNA panhandle were added to a fixed concentration of radiolabeled 5’ NCR (E) and 3’ NCR (F). Reaction mixtures were filtered through a nitrocellulose filter as mentioned above for the generation of binding profiles.

Since N protein showed different binding affinities for wild type vRNA panhandle and single strand RNA, we asked whether it has different binding modes for the two types of RNA substrates. Purified N protein was incubated with radiolabeled wild type panhandle RNA to form the N protein-panhandle complex. The complex was chased with increased input concentrations of either cold wild type panhandle RNA or cold thirty nucleotide long 5’ or 3’ NCR sequences (Fig 4A). It is evident that cold panhandle efficiently competed with radiolabeleld panhandle for binding to N protein. However, the cold 5’ and 3’ NCR sequences failed to do so. This suggests that N protein likely has different binding modes for vRNA panhandle and single strand 3’ or 5’ NCR sequences. In a similar experiment a preformed complex between N protein and radiolabeled 5’ NCR sequence was chased with either cold panhandle or cold 5’ or 3’ NCR sequences. Interestingly, both cold 5’ and 3’ NCR sequences efficiently competed with radiolabeled 5’ NCR sequence for binding to N protein. In comparison, the cold panhandle failed to compete with radiolabeled 5’ NCR for N protein binding (Fig 4B). The results from Fig 4B clearly demonstrate that 5’ and 3’ NCR sequences share a common binding mode for binding to N protein, which is different from the vRNA panhandle binding mode. Similar results were obtained from the competition experiment in which a preformed complex between N protein and radiolabeled 3’ NCR sequence was chased by either cold panhandle or cold 5 or 3’ NCR sequence (Fig 4C).

To further confirm the results of Fig 4A–4C, we asked whether a complex of N protein with 5’ or 3’ NCR can still bind to vRNA panhandle or vice versa. This simultaneous binding is possible only if N protein has different binding modes for vRNA panhandle and single strand 5’ or 3’ NCR. We incubated purified N protein with cold 5’ NCR sequence at a molar ratio of 1:100 of N protein:cold 5’ NCR to allow the complex formation. This complex was tested for the binding with hot vRNA panhandle. Using filter binding assay, a fixed concentration of hot vRNA panhandle was incubated with the increasing input concentrations of the complex. Reaction mixtures were filtered through nitrocellulose filter and the retention of hot vRNA panhandle on the filter was monitored. It is evident from Fig 4D that preformed N protein-5’ NCR complex was able to bind to the radiolabed vRNA panhandle, suggesting that N protein simultaneously binds to both the vRNA panhandle and 5’ NCR sequence. It must be noted that both free N protein and preformed N protein-5’ NCR complex bound to radiolabeled vRNA panhandle with similar affinity (compare Fig 4D with Fig 2 top panel). Similarly, a preformed complex between N protein and wild type vRNA panhandle was challenged for simultaneous binding to the radiolabeled 5’ or 3’ NCR. Fig 4E and 4F demonstrate that N protein bound to the vRNA panhandle through dsRNA binding mode simultaneously binds to 5’ or 3’ NCR sequence ssRNA binding mode. Again, the binding affinities of free N protein and N protein-panhandle complex with either 5’ or 3’ NCR are comparable (compare Fig 4E and 4F with Fig 2 bottom panels). Taken together, the data from Fig 4 shows that N protein has distinct binding modes for vRNA panhandle and single strand 5’ or 3’ NCR.

### Fluorescence binding

**Fig 5:**
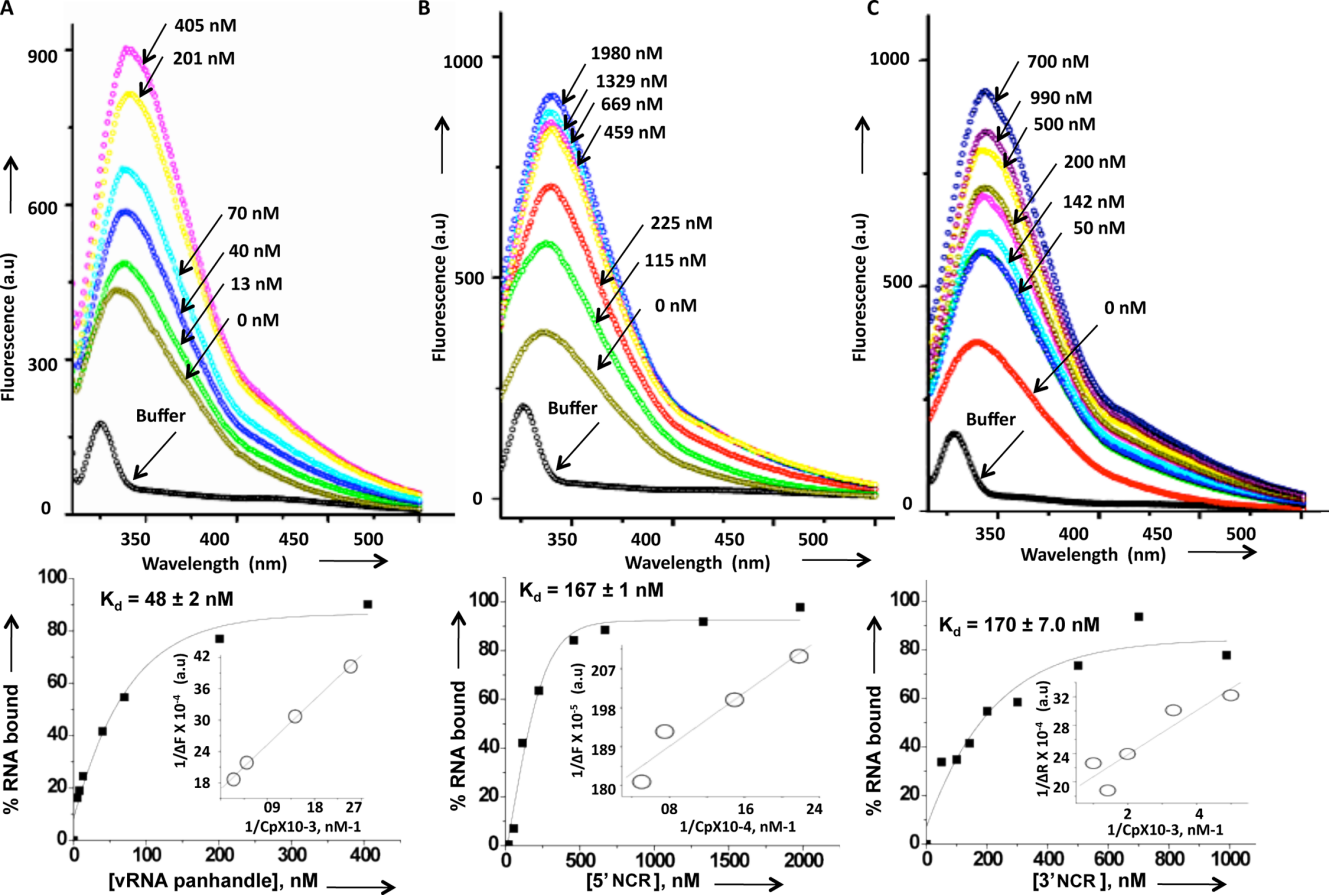
Fluorescence spectroscopic analysis for the association of CCHFV N protein with the vRNA panhandle, 5’ and 3’ NCR sequences. (A). As described in the Materials and Methods, a fixed concentration of N protein in RNA binding buffer was excited at 295 nm, and fluorescence spectrum from 300–500 nm was recorded. The fluorescence signal from binding buffer without N protein was subtracted from all the spectra shown in this panel. Increasing concentrations of the panhandle, shown by arrows, were added to the N protein and the fluorescence spectra (shown by different colors) were recorded. The fluorescence intensity value at 334 nm from each spectra was plotted verses input concentration of the panhandle to generate the binding profile for the calculation of K_d_ (shown at the bottom), as discussed in materials and methods. All fluorescence experiments were carried out at room temperature. (B and C). The fluorescence experiment shown in these two panels was carried out similar to the panel A, except the vRNA panhandle was replaced by 5’ NCR and the 3’ NCR in panels B and C, respectively. The increasing input concentrations of the 5’ and 3’ NCR are shown by the arrows. The corresponding binding profiles are shown at the bottom. The insets in the binding profiles show the double reciprocal plot for the calculation of ΔF_max_ that was used in Eq 4 for the calculation of K_d_ values, as mentioned in materials and methods.

We next used fluorescence spectroscopy to further confirm the binding affinity of N protein with the wild type vRNA panhandle and single strand 5’ and 3’ NCR sequences, as previously reported [29]. N protein has nine tryptophan residues per polypeptide. As shown in Fig 5, N protein generated a tryptophan fluorescence spectrum with an emission peak at 334 nm. The fluorescence emission peak of free tryptophan is at 350 nm. The 16 nm blue shift in the fluorescence emission peak of N protein compared to free L-tryptophan is due to the shielding of tryptophan residues in the folded N protein structure. The blue shift is also indicative of a hydrophobic microenvironment of tryptophan residues in the folded N protein structure. We monitored the change in tryptophan fluorescence spectrum of N protein with the addition of synthetic vRNA panhandle, 5’ and 3’ NCR sequences. The vRNA panhandle and NCR sequences were synthesized by *in vitro* T7 transcription. We observed a consistent increase in the tryptophan fluorescence quantum yield with a noticeable read shift in the fluorescence emission peak at each input concentration of vRNA panhandle, 5’ or 3’ NCR sequences (Fig 5). At saturating concentrations of vRNA panhandle the tryptophan fluorescence emission peak was red shifted by 7 nm compared to N protein lacking the vRNA panhandle. The similar peak shift observed for 5’ and 3’ NCR sequences was 5 nm. The red shift depicts the hydrophilic microenvironment of tryptophan residues due to RNA binding. It is likely that RNA binding induces a conformational change in the N protein, which exposes the buried tryptophan residues towards the protein surface facing the hydrophilic microenvironment. The increase in fluorescence intensity at each input concentration of RNA was used to generate the binding profile for the calculation of K_d_ (Fig 5D–5F), as discussed in materials and methods. The analysis of fluorescence data revealed that N protein bound to panhandle RNA, 5’ and 3’ NCR sequences with K_d_ values of 48±2 nM, 167±1 nM, 170±7 nM, respectively. These values are consistent with filter binding analysis and bio-layer interferometry (Fig 2).

### Binding of vRNA panhandle and single strand RNA induces a conformational changes in N protein

**Fig 6:**
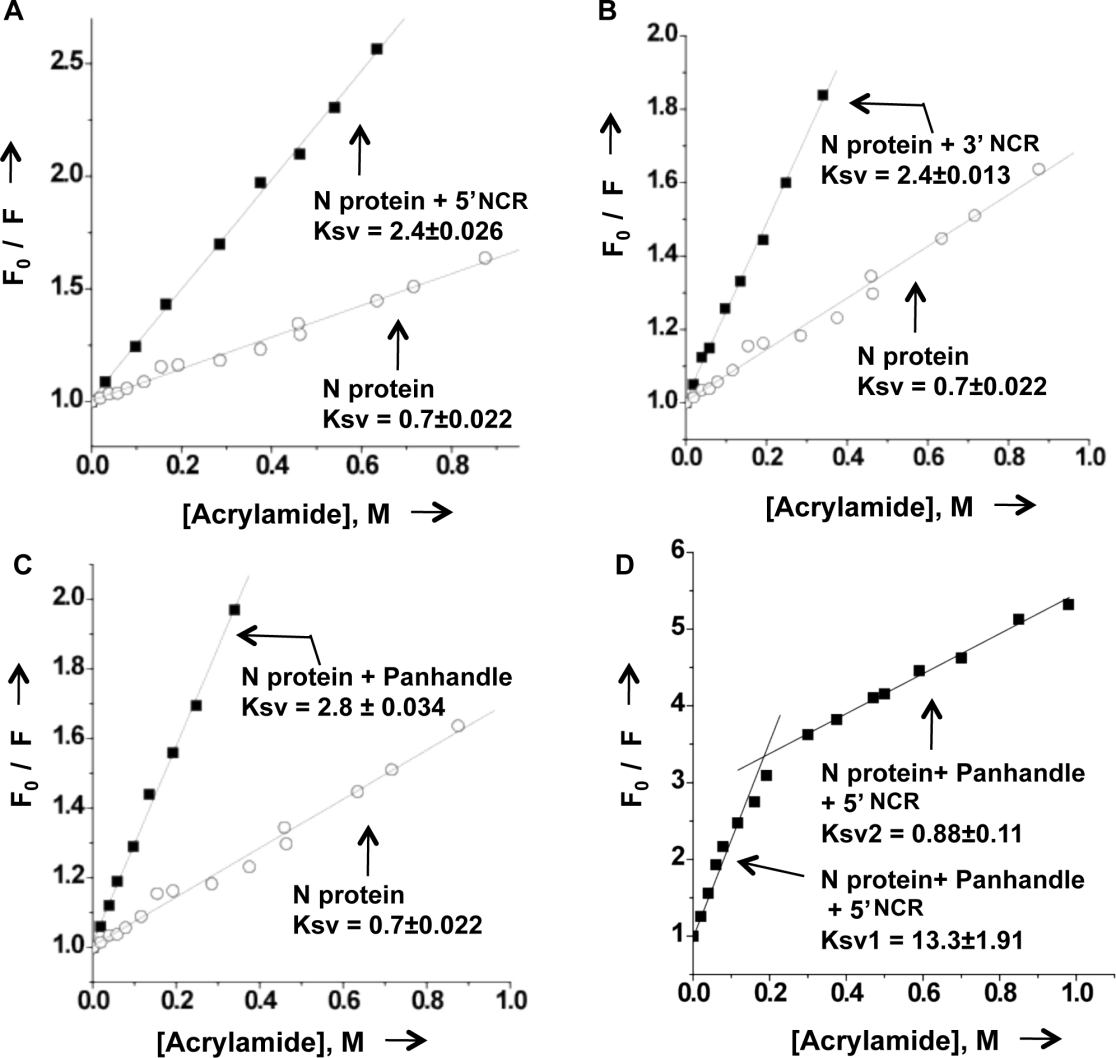
RNA binding induces conformation change in N protein. Stern Volmer quenching analysis of CCHFV N protein with the 5’ NCR (A), 3’ NCR (B), vRNA panhandle (C), and both 5’ NCR and vRNA panhandle (D). The analysis of N protein without RNA is shown by open circle in panels A, B and C. The analysis after the binding of N protein with the RNA of interest is shown by filled square. The Ksv values were calculated as mentioned in materials and methods.

To confirm the potential conformational changes in N protein due to RNA binding, we carried out stern volmer quenching analysis to monitor the accessibility of tryptophan residues of purified N protein to a neutral quencher (acrylamide) before and after binding to the RNA of interest, as described in materials and methods. As shown in Fig 6A, the tryptophan residues of N protein were accessible to acrylamide with a Ksv value of 0.7±0.022. However, after binding to 5’ NCR the Ksv value increased to 2.4 ±0.026, consistent with an increase in the accessibility of tryptophan to acrylamide quencher. Similar results were obtained when N protein formed a complex with the 3’ NCR (Fig 6B). The binding of N protein to vRNA panhandle resulted in the further increase of Ksv value (2.8±0.034), consistent with the further increase in the accessibility of tryptophan to acrylamide quencher due to panhandle binding. The observed increase in the accessibility of tryptophan to acrylamide is due to the induced conformational change in N-protein after RNA binding. The differential tryptophan accessibility due to the binding of vRNA panhandle and single strand 5’ or 3’ NCR sequences is consistent with the two distinct binding modes for the two types of RNA substrates. The increased accessibility of tryptophan residues to neutral quencher due to RNA binding (Fig 6) is consistent with the increased exposure of tryptophan residues to the hydrophilic surface environment due to RNA binding, evident from the red shift of tryptophan fluorescence peak in Fig 5. Interestingly, the simultaneous binding of N protein to both vRNA panhandle and 5’ NCR generated two Ksv values (Fig 6D). In the co-complex of N protein with both vRNA panhandle and 5’ NCR, a fraction of tryptophan residues were highly accessible to acrylamide, evident from very high Ksv value of 13±1.91 (Ksv1). Another fraction of tryptophan residues was poorly accessible to acrylamide, generating a Ksv value of 0.88±0.11 (Ksv2). This interesting observation (Fig 6D), suggests that N protein is in a unique conformational state when both vRNA panhandle and single strand 5’ NCR are simultaneously bound. It must be noted that *Ksv = K*_*q*_*[τ*^*o*^*]*, where *K*_*q*_ is the bimolecular quenching constant and *τ*^*o*^ is the excited state life time of the fluorophore in the absence of quencher. Thus the observed changes in the Ksv values due to changes in the *τ*^*o*^ cannot be ruled out.

**Fig 7:**
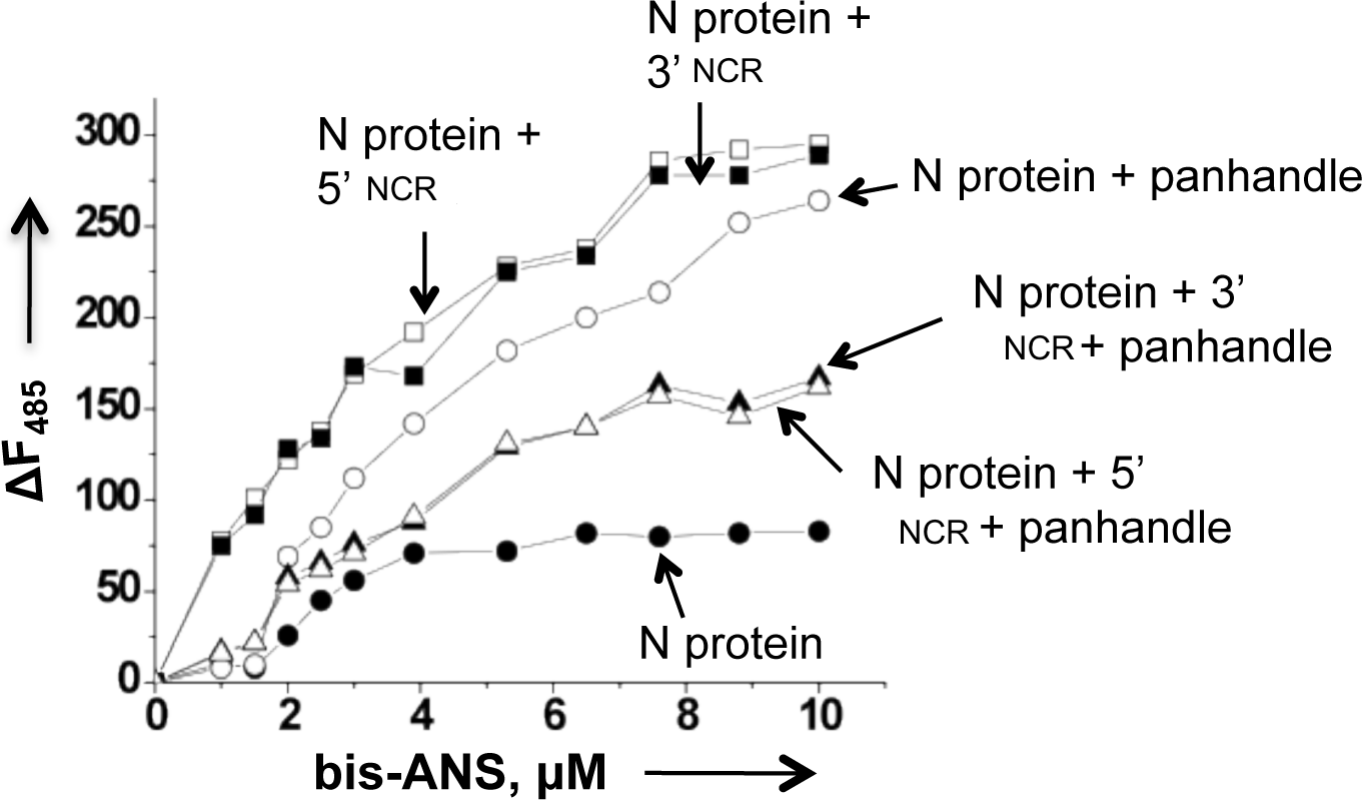
Probing the structural alteration in CCHFV N protein after binding to the RNA of interest using a fluorophore bis-ANS. Fluorescence titration of CCHFV N protein with hydrophobic fluorophore (bis-ANS) in RNA-binding buffer at room temperature under different conditions. The fluorophore was excited at 399 nm, and the emission was recorded at 485 nm. Shown are the titration curves of bis-ANS binding with either free N protein at a concentration of 150 nM (filled circle) or after incubation at room temperature for half an hour with 350 nM vRNA panhandle (open circle), 5’ NCR (open square), 3’ NCR (filled square), both 5’ NCR and vRNA panhandle (open triangle), both 3’ NCR and vRNA panhandle (filled triangle).

We next employed bis-ANS as probe to monitor the difference in conformational changes, if any, induced by the binding of vRNA panhandle, single strand 5’ or 3’ NCR or the simultaneous binding of both vRNA panhandle and single strand RNA. The increase in the fluorescence signal of bis-ANS observed upon association with N protein is due to the binding of probe to the hydrophobic pockets of the protein. The fluorescence signal of bis-ANS increased with similar high magnitude upon binding to the preformed complex of N protein with either 5’ or 3’ NCR (Fig 7). This suggests that a complex of N protein with both 5’ and 3’ NCR have similar hydrophobic binding sites available for bis-ANS binding, implying that two complexes are structurally similar. This again is consistent with the idea that both 5’ and 3’ NCR sequences bind through the same binding mode. In comparison, the bis-ANS binding sites were reduced in the complex formed between N protein and vRNA panhandle, evident from decreased bis-ANS fluorescence signal (Fig 7). The bis-ANS binding profile of N-panhandle complex is different for the complexes of N protein with 5’ or 3’ NCR (Fig 7), suggesting the complexes are structurally different. This observation further supports the two distinct binding modes for vRNA panhandle and single strand 5’ or 3’ NCR. To further confirm the observation that N protein is in a different conformational state when both panhandle and ssRNA are simultaneously bound, we monitored the binding of bis-ANS to a preformed complex of N protein with either panhandle and 5’ NCR or panhandle and 3’ NCR. Interestingly, the resulting complexes showed further decrease in bis-ANS binding and their binding profiles were similar (Fig 7). This confirms that simultaneous binding of panhandle and single strand RNA keep N protein in a unique conformational state.

### CCHFV N-protein does not unwind the RNA duplex after binding

**Fig 8:**
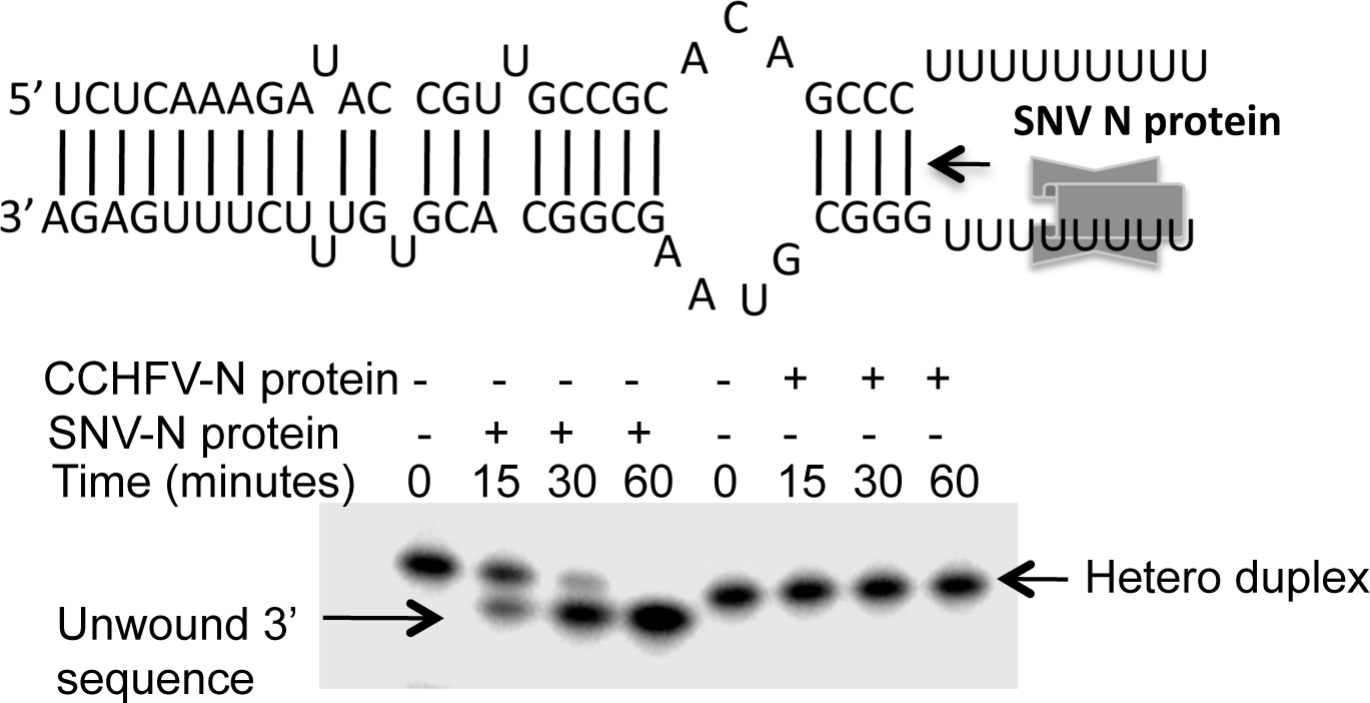
CCHFV N protein does not have RNA unwinding activity. An RNA heteroduplex formed between radiolabeled 3’ and cold 5’ UTR sequences of CCHFV S-segment vRNA is shown at the top. Both the UTR sequences contained a stretch of “U” residues. SNV N protein unwinds the heterodupplex from 5’ to 3’ direction and requires a single strand RNA sequence to initiate unwinding. The heteroduplex shown at the top was incubated with either SNV N protein of CCHFV N protein for increasing time intervals. The mixture was fractionated on 10% SDS-PAGE to examine the release of radiolabled 3’ UTR sequence (see materials and methods for details).

Since N protein bound the vRNA panhandle with high affinity, we asked whether panhandle remains double stranded after association with N protein. To rule out the possible melting of vRNA panhandle we examined the RNA dissociation activity of N protein, as previously reported [37]. We have previously reported that SNV N-protein has RNA chaperone activity and unwinds the double strand RNA from 5’ to 3’ direction in ATP independent manner [37,38]. As shown in Fig 8, the SNV N-protein requires a single strand RNA sequence to initiate the duplex unwinding. We synthesized the 5’ and 3’ NCR sequence of CCHFV S-segment vRNA containing a stretch of uracil residues (Fig 8). The radiolabeled 3’ NCR was incubated with the cold 5’ NCR to form a panhandle structure, as mentioned in materials and methods. The radiolabeled panhandle was gel purified and incubated with either CCHFV N-protein or SNV N protein as positive control, for increasing time intervals. It is evident from Fig 8 that SNV N protein unwound the panhandle structure, observed by the release of radiolabeled 3’ sequence from the heteroduplex. The CCHFV N protein failed to unwind the panhandle, suggesting that double strand RNA does not melt after binding to CCHFV N protein.

## Discussion

We examined the interaction of bacterially expressed and purified CCHFV N protein with the synthetic vRNA. It is evident from Fig 1 that N protein binds with same affinity to both the CCHFV S-segment vRNA and G*luc* RNA harboring the vRNA termini, suggesting that non-coding vRNA termini are sufficient for high affinity binding. Deletion of either 5’ or 3’ terminus impacted the binding, indicating that panhandle structure might be important for the high affinity binding with the N protein. Further analysis by two independent experimental approaches confirmed that N protein bound to the panhandle structure, composed of 32 nucleotides from both 5’ and 3’ termini of S-segment vRNA, with same affinity as full length vRNA (Kd ~42 nM). Further characterization demonstrated that terminal nucleotides of the panhandle play an important role and the primary sequence of the panhandle is required for high affinity binding (Fig 3). Unlike hantavirus N protein, the CCHFV N protein did not unwind the vRNA panhandle after binding (Fig 8). Interestingly, N protein also bound to the single strand 5’ or 3’ NCR (Fig 2) with same weaker affinity as single stranded nonviral RNA (kd~121 nM) (Fig 3). The competitive binding (Fig 4) clearly demonstrated that N protein has distinct binding modes for both vRNA panhandle and single strand RNA (ssRNA). Alterations in the tryptophan fluorescence signal revealed a conformational change in N protein due to RNA binding (Fig 5). This analysis further demonstrated the distinctive nature of panhandle and ssRNA binding to the N protein. Both the Stern Volmer quenching analysis and bis-ANS binding (Figs 6 and 7) confirmed the induction of distinctive conformational changes in N protein due to the binding of structurally distinct vRNA panhandle and ssRNA through two distinct binding modes. In addition, both the spectroscopic approaches revealed that N protein attains a unique conformational state when simultaneously bound to both vRNA panhandle and 5’ or 3’ single strand NCR.

**Fig 9:**
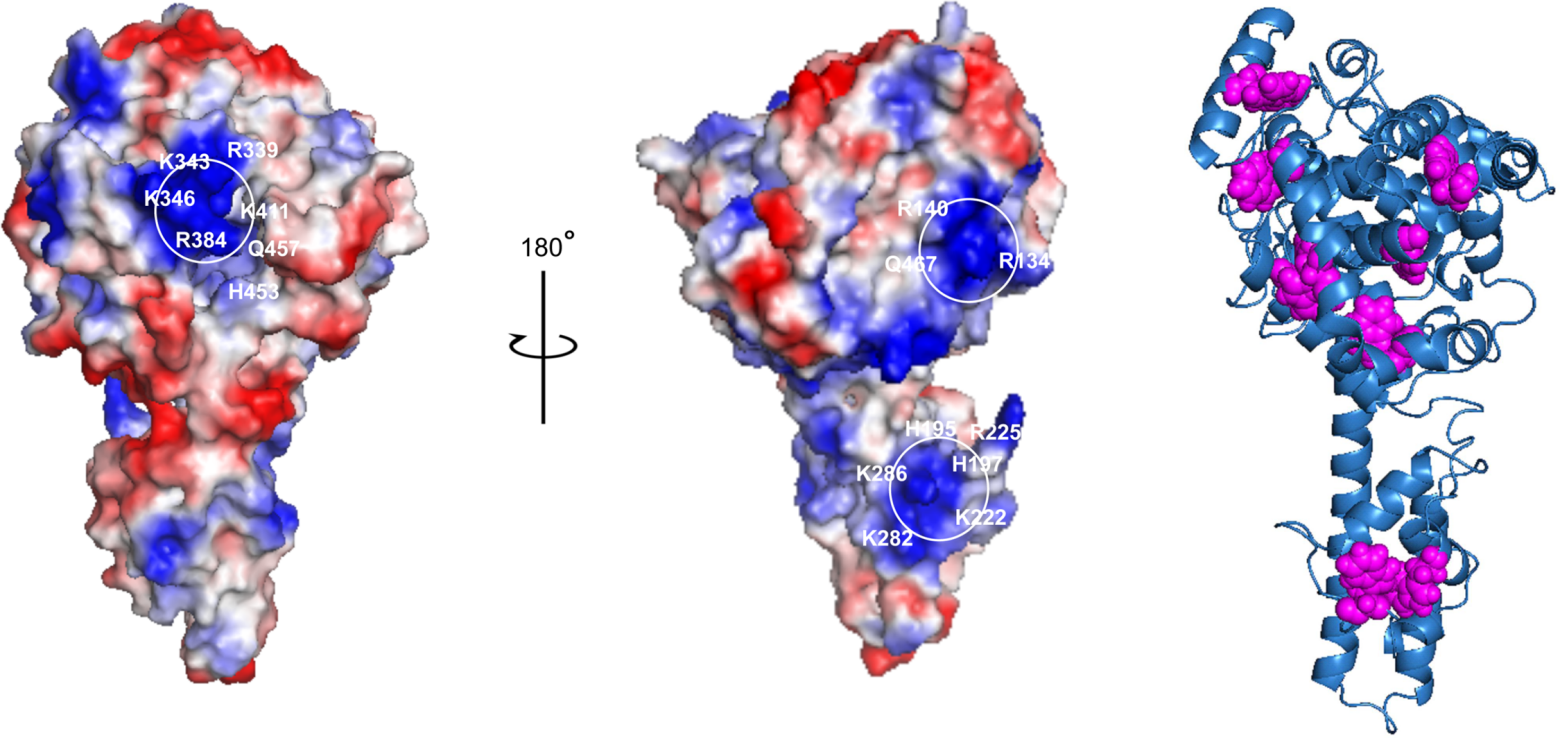
Modeled 3D structure of CCHFV N protein. Potential RNA-binding regions of CCHFV N protein. The electrostatic surface potential of N protein was visualized by PyMol. The positive surface is colored blue and the negative surface is colored red. The isolated positively charged surfaces constituted by residues (K339, K343, K346, R384, K411, H453) and (R134, R140, Q467) and (H195, H197, K222, R225, K282, K286) may be involved in the formation of hypothetical binding pockets for the vRNA panhandle and single strand RNA. A cartoon of CCHFV N protein in which tryptophan residues are highlighted as pink spheres is shown at the right.

Recently the X-ray crystal structure of CCHFV N protein (strain YL04057, PDB code 3U3I) demonstrated a racket-shaped overall structure with distinct “head” and “stalk” domains. The structure significantly differs from other nucleoproteins reported from other negative-sense RNA viruses. We modeled the 3D structure of CCHFV N protein (strain 10200). Although, the primary sequences of these two strains are slightly different, the modeled structure of strain 10200 is very similar to the X-ray structure of strain YL04057 (Fig 9). The electrostatic surface potential of N protein, generated by PyMol viewer software tool, predicted three potential RNA-binding regions based on the positively charged grooves at the surface of N protein structure. This observation supports the potential existence of distinct vRNA panhandle and ssRNA binding sites in CCHFV N protein. The high positive change of the putative RNA binding site and weaker impact of salt on RNA binding affinity suggests that amino acids away from the putative RNA binding pockets may also play critical role in the RNA binding. The potential roles of two distinct RNA binding modes of CCHFV N protein in the virus life cycle remain unclear. The N protein from both CCHFV and hantavirus has been reported to interact with viral RdRp [12,13]. Also, the panhandle structure has been reported to function as a promoter for the Bunyaviridae RdRp [39]. In addition, we previously reported that hantavirus N protein specifically binds to the vRNA panhandle. Since N protein (hantavirus and CCHFV) binds to both the vRNA panhandle and the RdRp [12,33], it is possible that panhandle binding site of N protein may play a role in the replication of viral genome in conjunction with RdRp.

The negative sense Bunyaviridae genome remains associated with the viral N protein to form nucleocapsids that serve as templates for the synthesis of viral mRNA and replication of the viral genome by the RdRp. The viral genome must harbor cis-acting signals that are specifically recognized by the N protein to ensure the selective encapsidation. Numerous *In vitro* binding studies have previously used bacterially expressed and purified *Bunyaviridae* N protein to examine its specific binding with the synthetic viral genomic RNA [40–44]. These studies have suggested that *Bunyaviridae* N protein appears to preferentially interact with a target site near the 5’ end of the vRNA, proposing that vRNA 5’ terminus might have cis-acting encapsidation signals. However, the recent studies have proposed that nascent vRNA is co-transcriptionally encapsidated. Recently, the x-ray and cryo-EM studies of La Crosse virus have provided insights about the location of important functional sites in the Bunyaviridae RdRp [45]. The Bunyaviridae RdRp has distinct binding sites for 5’ and 3’ termini of vRNA. The catalytic site is located towards the center of the protein. Identification of distinct template and product exit tunnels has led to the proposition of a novel model for template-directed replication of viral genome by the RdRp molecule [45]. According to this model, the 5’ terminus of nascent vRNA is encapsidated by the incoming N protein and RdRp as it exits through the product exit tunnel of the RdRp molecule. We observed that deletion of 3’ NCR in the G*luc* RNA 3’ del (Fig 1) showed a cooperative binding behavior with the N protein. It is possible that positive cooperative binding of N protein to the single strand 5’ NCR might have a role in the initiation of vRNA encapsidation. With the completion of transcription cycle the nascent vRNA is fully encapsidated, and the 5’ and 3’ termini are bound to their respective binding sites of the RdRp, preventing the formation of panhandle structure. We propose that *Bunyaviridae* RdRp might exist in transcriptionally active and inactive states. In transcriptionally inactive state, the 5’ and 3’ vRNA termini might exit their respective binding pockets of the RdRp and undergo base pairing to form the panhandle structure, which is specifically recognized by N protein to stabilize the inactive state of the replication complex. Formation of such transcriptionally inactive complex is possible during the assembly stage of virus replication when genome replication is halted. Transcription activation might lead to the dissociation of N protein from the panhandle, enabling its melting and subsequent incorporation of 5’ and 3’ termini to their respective binding pockets of the RdRp, triggering the transcription initiation. It is equally probable the N protein-panhandle interaction might be involved in the incorporation of nascent nucleocapsids into new virions during the virus assembly. N protein-panhandle interaction might play other roles in CCHFV replication that are unknown at present.

## Conclusion

CCHFV nucleocapsid protein has two distinct binding modes for double and single strand RNA. Through double strand RNA binding mode, the nucleocapsid protein preferentially binds to the vRNA panhandle. However, the nucleocapsid protein does not discriminate between viral and non-viral RNA molecules through the single strand RNA binding mode. Binding of both vRNA panhandle and single strand RNA induce a conformational change in the nucleocapsid protein. Nucleocapsid protein remains in a unique conformational state when both vRNA panhandle and single strand RNA molecules are simultaneously bound to it. Although the role of dual RNA binding modes in the virus replication cycle is unknown from our study, their involvement in the regulation of CCHFV replication in conjunction with RdRp and host derived RNA regulators cannot be ruled out.

## Supporting information

S1 Fig (A): A 6% acrylamide urea gel showing four RNA molecules that were purified by gel extraction using denaturing PAGE (B): Binding profiles for the interaction of N protein with RNA molecules shown in panel A.
(TIF)

S2 Fig A 14% acrylamide urea gel showing three radiolabled RNA molecules that were tested for N protein binding using the filter binding assay (A). Binding profiles for the interaction of N protein with the 5’ NCR (B), 3’NCR (C) and wild type panhandle (D) form panel A are shown. The binding profiles were generated by filter binding assay.
(TIF)

S3 Fig (A). A 14% acrylamide urea gel showing six radiolabled RNA molecules. The sequence of these RNA molecules is shown in Fig 3 (B). Binding profiles for the interaction of N protein with the four RNA molecules from Panel A. The binding profiles for RNA molecules from lanes five and six of panel A are shown in Fig 3.The binding profiles were generated by filter binding assay.
(TIF)
