# Supplementary figures and images for: Crimean-Congo hemorrhagic fever virus nucleocapsid protein has dual RNA binding modes
Source: PLoS One. 2017 Sep 18;12(9):e0184935. doi: 10.1371/journal.pone.0184935 (PMC5602631; doi:10.1371/journal.pone.0184935)

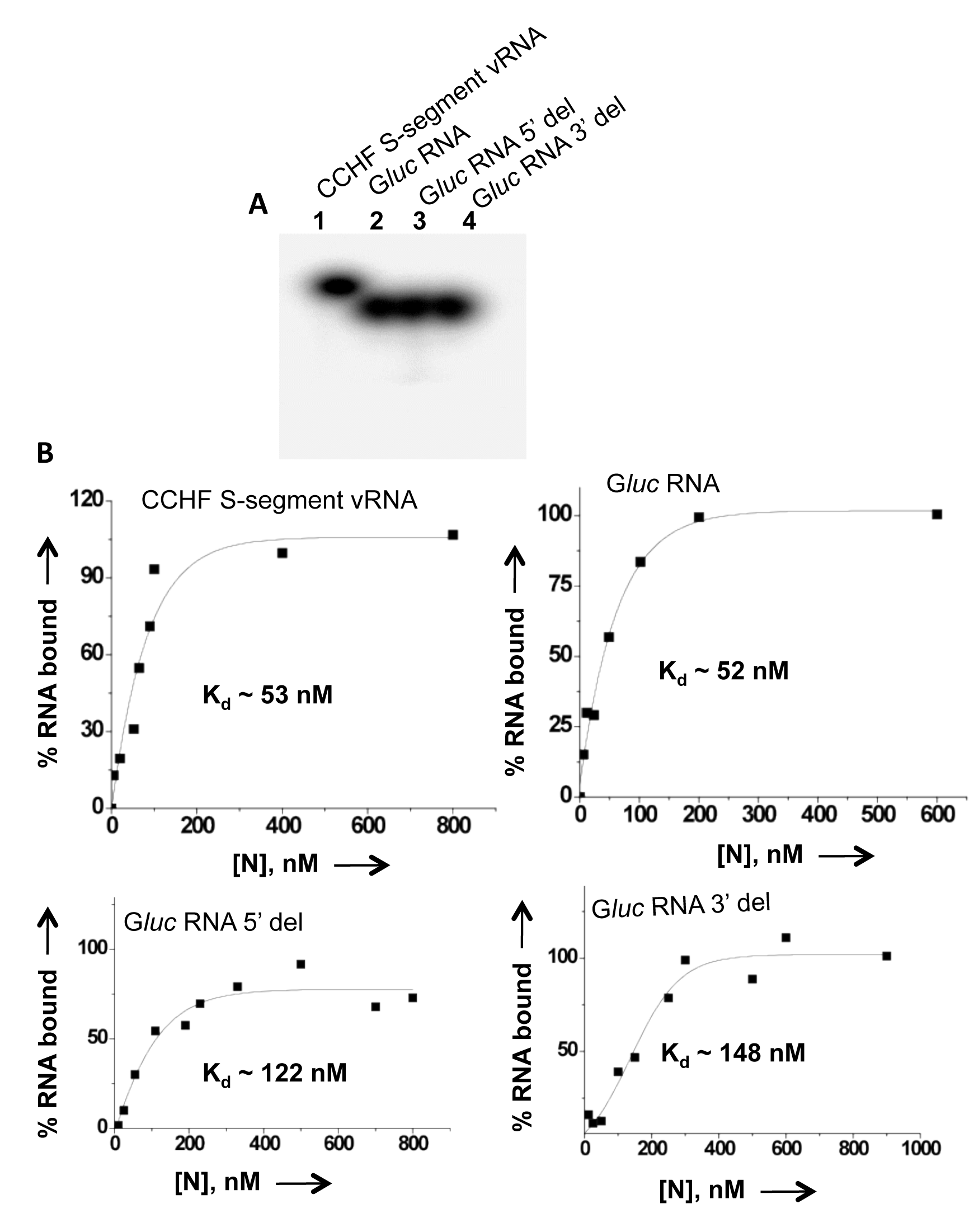

Supplement: S1 Fig — (A): A 6% acrylamide urea gel showing four RNA molecules that were purified by gel extraction using denaturing PAGE (B): Binding profiles for the interaction of N protein with RNA molecules shown in panel A. (TIF) [file pone.0184935.s001.tif]

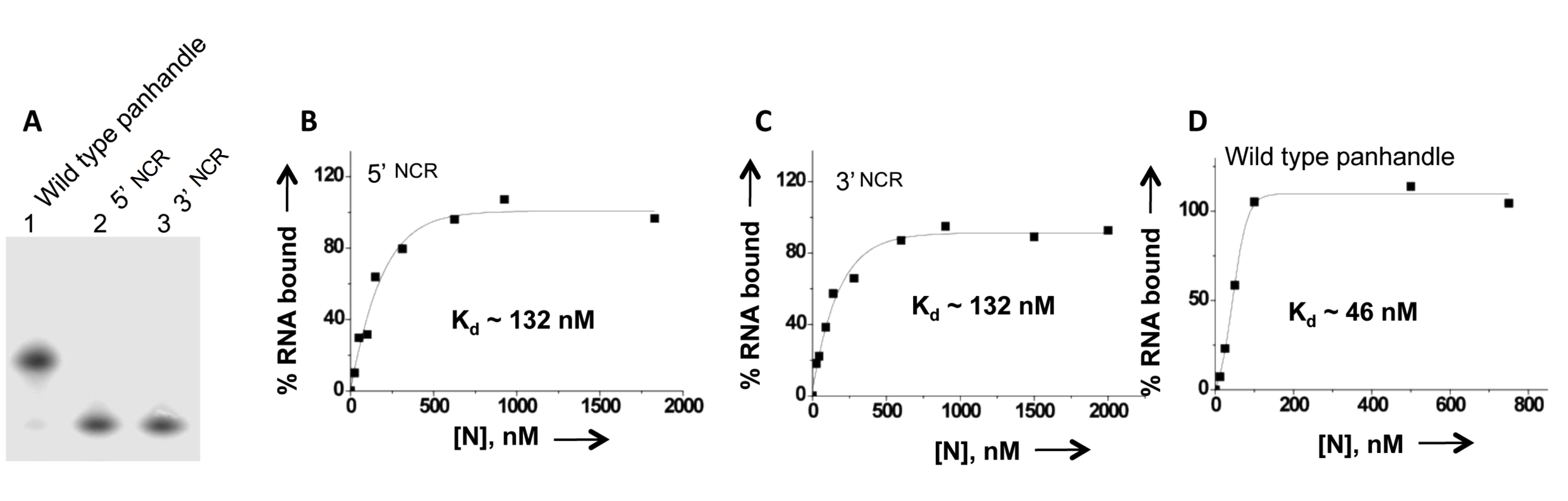

Supplement: S2 Fig — A 14% acrylamide urea gel showing three radiolabled RNA molecules that were tested for N protein binding using the filter binding assay (A). Binding profiles for the interaction of N protein with the 5’ NCR (B), 3’NCR (C) and wild type panhandle (D) form panel A are shown. The binding profiles were generated by filter binding assay. (TIF) [file pone.0184935.s002.tif]

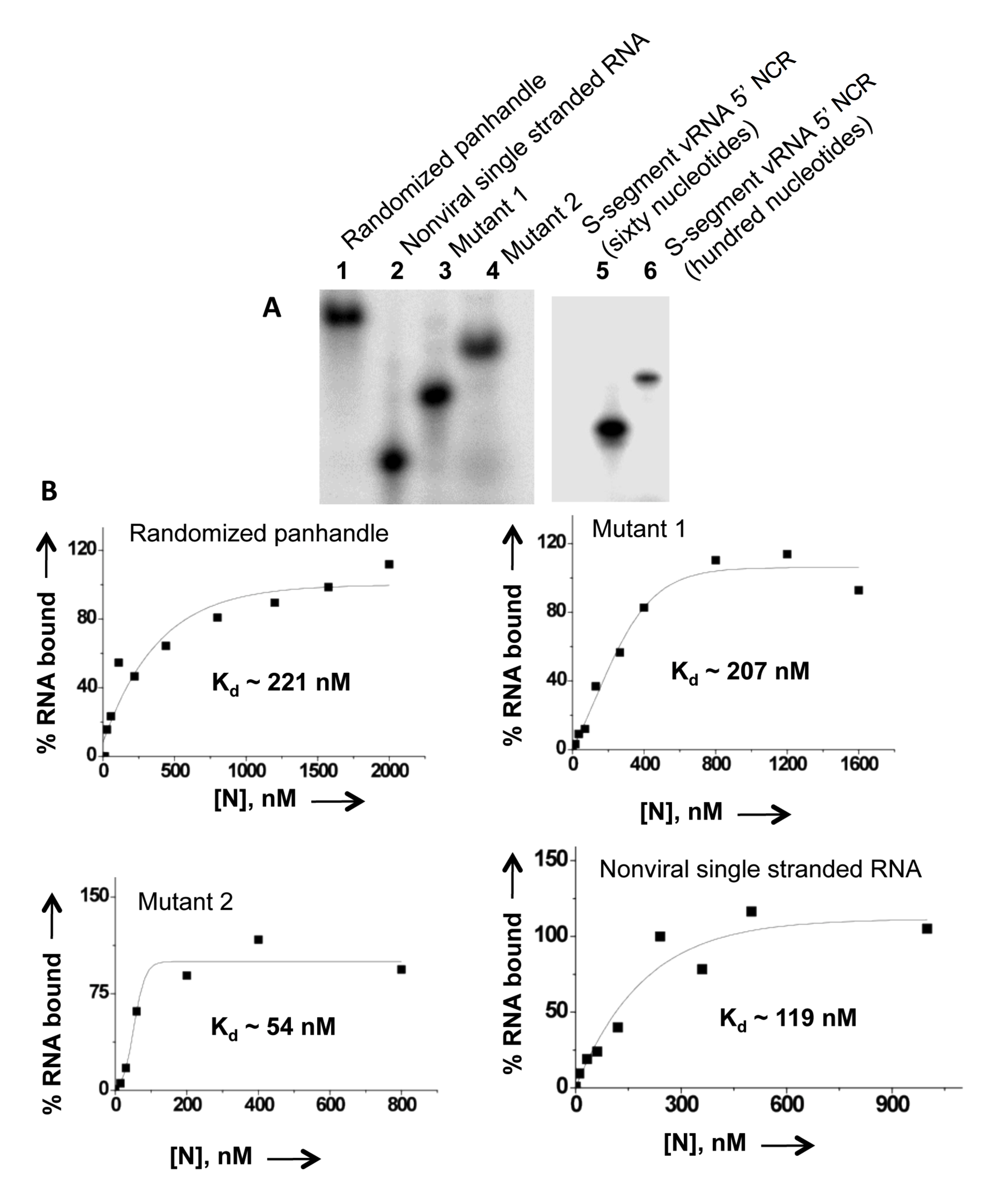

Supplement: S3 Fig — (A). A 14% acrylamide urea gel showing six radiolabled RNA molecules. The sequence of these RNA molecules is shown in Fig 3 (B). Binding profiles for the interaction of N protein with the four RNA molecules from Panel A. The binding profiles for RNA molecules from lanes five and six of panel A are shown in Fig 3.The binding profiles were generated by filter binding assay. (TIF) [file pone.0184935.s003.tif]
